# Implementing standardized cancer patient pathways (CPPs) – a qualitative study exploring the perspectives of health care professionals

**DOI:** 10.1186/s12913-019-4413-6

**Published:** 2019-08-16

**Authors:** Sara Delilovic, Henna Hasson, Mårten Åhström, Mia von Knorring

**Affiliations:** 1 Center for Epidemiology and Community Medicine, Stockholm County Council, Stockholm, Sweden; 2 PROCOME research group, Medical Management Centre, Karolinska Institutet, Stockholm, Sweden

**Keywords:** Standardized pathways, Cancer, Waiting time, Implementation

## Abstract

**Background:**

Many countries have implemented standardized cancer patient pathways (CPPs) to reduce waiting times in cancer care and to ensure timely and quick diagnosis as well as treatment. Yet, no studies have explored the implementation process as perceived by the health care professionals working in the CPPs. The aim of this study is to explore the experiences of health care professionals (HPCs) involved in the CPPs.

**Methods:**

A descriptive qualitative design was adopted. Thematic analysis was applied to individual interviews conducted in 2016–2017 with 58 participants working in six different CPPs in Sweden’s largest region, covering care for around 2.3 million inhabitants.

**Results:**

In general, the health care professionals had a positive attitude towards the implementation of the CPPs. Our findings showed that the CPPs require close collaboration, both between and within different health care professional groups and units, something that was not always probable due to differences in resource capacity. Better dissemination to all relevant professionals, better conceptualization, and equivalent opportunities in terms of resources were identified by the respondents as being important yet lacking in practice. The analysis showed possible negative effects of the CPP, such as crowding-out on other patient groups.

**Conclusion:**

The CPPs were introduced to address challenges with long waiting times and unequal cancer care. By exploring the experiences of health care professionals involved in the implementation of CPPs, our findings show challenges with multi-level coordination and collaboration, policy dissemination, and resource constraints. The analysis also showed that the implementation of CPPs risk being accompanied by unintended effects such as longer waiting times for other patients and patient groups in need of the same health care resources. The results shed light on and contribute to an understanding of the challenges, opportunities and ways forward.

## Background

Long waiting times for health services has been a politically urgent and prominent problem in many countries for decades, in both primary and secondary care [1]. Issues with waiting times arise as a result of complex interactions between demand and supply, and are not solely dependent on the supply side. The issue with long waiting times is of significance, as it may result in inequality in access to health care and generates disutility for patients in need of care [1, 2]. Thus, even within publicly funded health systems, where access does not depend on the ability to pay, there is no guarantee of equal access to health care services [1]. Cancer care is no exception. Many countries must deal with problems of long waiting times in cancer care [1]. Considering survival rates, trends show increases, but persistent differences exist between countries [3]. These differences lead to avoidable premature death and have pushed cancer control strategies into the political agenda. For Sweden, a country with one of the highest survival rates in the world, no major improvements in waiting times for patients in cancer care have been found during recent years. Furthermore, great differences, both between and within geographical areas, cancer forms, and sex, have been found [4–6].

As a response, a national policy was introduced by the Swedish government to address the challenges related to the long waiting times in cancer care. In 2015, standardized cancer patient pathways (CPPs) were launched to reduce waiting times in cancer care, reduce regional differences and provide a more equal footing to ensure better quality. The sought-after effect is to make cancer care more available and to increase patient satisfaction [7–9]. The policy was highly influenced by similar policy launched in Denmark in 2008 and in Norway in 2015 [10–14]. Furthermore, similar types of initiatives have been launched in other countries; in 2000, the UK implemented the so-called Two-Week wait rule for cancer referral as a part of their National Cancer Plan [15, 16]. Previous research shows that the implementation of the CPP has provided more timely diagnosis and treatment of patients with cancer [17] Results show higher survival rates and lower mortality for symptomatic patients with cancer diagnosed through primary care [13]. Nevertheless, changes in survival and mortality rates and the effects of the implementation of the CPP should be understood as a combination of actions taken in the implementation process. It is difficult to conclude which component of the CPP has had the greatest impact and led to the observed changes [17]. Implementation process has been described as unpredictable and complex, where numerous factors, in addition to the content of change influence the outcome and process. Leadership, characteristics of the organization, culture and context in which the process take place are some factors influencing and impacting implementation [18, 19].

### Patient Pathways

Patient pathways are often understood as clinical pathways operationalized as “standard packages” of procedures or measures of health care and they are based on medical guidelines and a set of processes of care that are likely to achieve desirable health outcomes [20]. The Swedish national policy includes a set of clinical guidelines for each cancer diagnosis, which lists and delineates specific criteria for symptoms that may raise suspicion of cancer. In addition, each cancer diagnosis has guidance manuals for diagnostic entities and referrals that are required in order to make a diagnosis (e.g., a visit to a medical specialist, radiology assessment, or pathological analysis) [8, 12, 13]. All investigating phases have an assigned maximum time-scale. Time-scales are based on optimal value-creating times for patients and vary between diagnoses. These give an indication of how much time is appointed to each necessary step in the diagnostic process, before a cancer diagnosis can be set. A CPP starts at the point when there is well-founded suspicion, either symptomatically expressed by the patient or by clinical evidence and ends with the start of the first treatment [8]. For a patient a CPP can take different forms as symptoms are diverse and may evolve over time, therefore, referral for a CPP can start either in primary or secondary care. Once a patient is referred to a CPP, all diagnostic and treatment procedures will be promptly organized in well-defined processes, such as clinical investigation and treatment [12].

### CPPs in the Swedish health care system

The Swedish health care system is highly decentralized. The central government is responsible for the overall health policies, while the local regions (County Councils) are responsible for the provision of health care to the citizens in their respective geographical areas. They operate autonomously and are governed by elected political representatives and are administered by officials [8, 21]. The general practitioners (GPs) in primary care often form the front line in health care and, in the context of CPPs, they play a vital role as “gatekeepers” to specialized care [8]. The CPPs have a multidisciplinary structure, involving health care professional in primary care and specialist care who individually and jointly hold responsibility for the patient’s continuity of care. With the multi-structure approach and broad stakeholder involvement, the CPPs require close collaboration and more integrated work, a potential challenge previously identified [8, 9, 22]. Nevertheless, the aim of the reform was to create new efficient ways of working with cancer processes, rather than assigning health care professionals with new tasks.

So far, no studies have qualitatively explored how the implementation process of the CPPs were perceived by health care professionals. In this study the aim is to explore the experiences of health care staff involved in the CPPs.

## Methods

### Design

A descriptive qualitative design was adopted. This design is considered valuable, particularly when exploring areas not widely studied, and it was judged to be the most suitable approach with respect to the research question [23, 24].

### Study setting

With the launch of the first national cancer policy in 2009, the government initiated the establishment of six regional cancer centers (RCCs) [6, 8]. The role of RCCs is to formulate and implement a plan for the healthcare region’s work with preventative measures and early detection of cancer. RCCs work long-term with different cancer strategies and aid in the coordination of multi-professional collaboration between and within health care professionals to increase quality of care, improve health outcomes and use health care resources efficiently. With the implementation of CPPs in Sweden, the RCCs, together with health care professionals and patient representatives, were assigned the duty of forming and designing the implementation. This was completed with a “bottom-up” adaptive approach, allowing health care professionals and patient representatives to influence the design and structure of the implementation. The reform was initiated by the central government but the CPPs were developed by medical specialist and multi-professionals care teams [8, 25]. The implementation was conducted with an adaptive approach, allowing strategies to be modified, revised and altered to meet the needs of local circumstances, contexts and needs [26].

As the implementation of the CPPs progressed, a new temporary administrative role of coordinator was established to better enhance the structure of referral routes for the patient pathways, situated at either hospital or hospital department level. [6, 8, 22]. Initially the measures taken in the CPP, for example lead-times (time appointed to each necessary step in the diagnostic process) were registered in classification codes in the electronical medical system. Health care professionals did not use the same medical record system and it was difficult to trace the patient and assure correct registration of the lead-times. Throughout the implementation, the platform INCA was established and used by all health care professionals to overcomes these barriers. To facilitate shorter lead-times, the investigatory units had pre-reserved slot that were unbookable for other patients than those in a CPP.

The CPPs began in 2015 with 5 diagnosis groups: acute myeloid leukemia, head and neck cancer, esophageal and gastric cancer, prostate cancer, and cancer of the bladder and urinary tract. Later, in 2016, 13 new cancer diagnosis groups were added among these were: other myeloma, lung cancer, brain tumors, cancer of unknown primary tumor and cancer of the bile disorders. One diagnosis group can include more than one cancer diagnosis.

For this study we have chosen to study the implementation process in Stockholm County. Stockholm County Council (SCC) is one of Europe’s largest healthcare providers, covering around 2.3 million inhabitants. SCC offers specialist care at university hospitals, have the best trained staff and the largest expenditure of the total county budget is within health care. [27].

For the purpose of the study, six diagnosis groups were chosen by the RCC Stockholm County: *Upper gastrointestinal cancers (stomach, small bowel cancer, pancreatic cancer, liver cancer and gallbladder cancer), anal cancer, gynecological cancer (ovarian cancer and cervix cancer), brain tumor, lung cancer, and myeloma*. The rationale for choosing these was to gain a heterogeneous variety of diagnosis. The six CPPs differ in and among others: *diagnosis complexity* (some cancer types are more difficult to diagnose), variety *of provider* (some are characterized by larger degrees of private providers), *incidence,* and *mortality* [28]. This enables the exploration of commonalities across diverse diagnoses and identifies features that cut across cases and contexts [29, 30]. In order to understand the entire process, we included all organizations involved in the CPPs, i.e., primary care units, specialist care units, and investigatory units (radiology, pathology, endoscopy and oncology).

### Participants

Within each of the six CPPs, a snowballing recruitment strategy was used to achieve a purposive sample of participants [31]. In the first step, the regional cancer center steering group was contacted to identify key persons working with the CPPs, 14 individuals were identified, whereof one declined participation. These initial respondents were then asked to recommend other key health care professionals within each specific CPP. In this step, recruitment was completed, directly after the conducted interview, on site, with the initial respondent or the respondent gave contact information to others colleagues. Participants were purposively recruited, on the basis that they were compatible of addressing the research question and directly involved in the practice of providing CPP services. All respondents were involved in CPPs, either clinically or administratively. To strengthen the credibility, we aimed to ensure representativeness by professional background [29, 32]. The distribution by professional background was 50% coordinator/administrator/nurse and 50% physicians. All respondents were health care personnel and had functions in the CPPs such as; resident physicians, internet physicians, senior specialists, surgeons, nurses, enrolled nurses and coordinators. Respondents who refrained from participating did so because of a lack of time, were not interested, or had no function related to the CPPs.

The final sample was comprised of 58 health care professionals working in the six cancer pathways and their investigatory units. The number of respondents by diagnosis and investigatory unit were: 7 upper gastrointestinal cancers, 7 lung cancer, 7 endoscopy, 7 primary care, 5 oncology, 5 gynecological cancer, 5 radiology, 5 brain tumor, 4 pathology, 4 myeloma, and 2 anal cancer.

### Data Collection

Data were collected from January 2017 to October 2017 using individual semi-structured interviews. To foster reflexivity and reduce bias, two researchers were included in data collection [23]. All interviews were carried out by interviewers with extensive experience of interviewing (two being the first and third authors). The interviewers had no relationship with the participants. Participants were initially contacted by phone or email and invited to participate. Two interview guides were developed; one for physicians, and one for coordinators/nurses/administrative staff. The reason for this was mainly due to their different functions in the CPPs. Domains for the interview guide were generated from previous research within the field [6, 8]. The focus was to understand how the implementation evolved in the participants’ own settings. The interview guides contained questions that addressed*: perceptions of the implementation process, experiences of working with the CPPs, capability to work with the CPPs, and barriers and facilitators.* Interviews lasted, on average, about 45 min. Data was collected and analyzed until saturation was reached, which means no new information or themes were observed in the data.

### Data analysis

All interviews were conducted in Swedish, digitally recorded and transcribed verbatim. A thematic analysis was conducted [33]. The analysis was guided by the different phases of thematic analysis, based on the techniques of systematically identifying themes across the data set, reviewing themes, and assuring coherent patterns revealed in the data. Patterns were identified through a rigorous process of data familiarization, data coding and theme development. Analysis was completed with an inductive approach, where themes were generated by the content of the dataset. All interviews were transcribed verbatim. The analysis was performed by the first author in collaboration with the co-authors, it was then refined, and issues and reflections were discussed among the team. The data were analyzed as a whole and not stratified by professional category or cancer diagnosis groups. To strengthen the validity, quotes were used to illustrate the findings and to show the logic behind the interpretation of data [29].

### Ethical considerations

Ethical clearance was obtained from the regional ethics board of Stockholm County (2017/1328–31). Written consent was obtained from all participants after introducing them to the study. Information was provided to them about their anonymity and secure data processing. Respondents were also informed about their right to withdraw their participation at any time without further explanation. In the presentation of the findings, all quotes are anonymized.

## Results

In the analysis, four main themes describing different aspects of the health care professionals’ experiences of the CPPs were identified. These themes were related to (1) *Readiness for implementation, (2) Intrinsic beliefs and perceptions of the policy’s value, (3) Complexity of joint action between levels in health care,* and (4) *Priorities and unintended effects.* Under each of these themes, a varying number of subthemes were identified that describe the staffs’ experiences of the CPP implementation process. Below, each theme and its subsequent subthemes are presented. The themes and subthemes are illustrated with quotes that are poignant and representative of the findings.

### Readiness for implementation

#### Lack of knowledge and conceptualization of the CPPs

Despite the intention of promoting a bottom-up process, the respondents expressed shortcomings regarding involvement in the implementation as well as a lack of adequate information on how to work and adapt components of the policy to suit existing working methods. Respondents reported being aware of the new reform, but fewer expressed having in-depth knowledge about the characteristics of the CPPs and the conceptualization of the new reform and the components that it encompassed. Information was said to have reached a select number of individuals who then failed to anchor and communicate these to the rest of the work team.

*“Nobody really knew anything, it felt a little bit like ... It just came, suddenly, from nowhere. And when it was new, then it was just suddenly that we had to start with this without having received any information or anything.” (Upper gastro 5).*

Issues highlighted were that information was not provided in a sufficient way to all relevant health care professionals, either within or outside their own setting. The dissemination of information to the health professionals, particularly in primary care, was said to be inadequate and, due to this, the initial phase of the implementation was to some degree regarded as being unsuccessful.

*“They completely skipped that educational part [to primary care], it was just put on the county council to try to fix it in the best way, it was a bit sad … if you properly think through when you make such a big change, you know that you have to build the foundation first, and make sure it is stable, do not build the house and put it on fragile ground, sadly it turned out a bit like that.” (Primary care 5).*

*“I think the awareness among referral units could increase, because, sometimes you get a referral where you feel this is a patient who meets the criteria for a CPP, and when you reconnect to the referral unit, they had never heard of the CPPs.” (Endoscopy 2).*

### Intrinsic beliefs and perceptions of the policy’s value

#### A means towards equal and improved cancer care

The policy was considered to be an important means towards improving cancer care and it was described as having a beneficial value, both for the health care system and for individuals under cancer investigation, not least in terms of equal cancer care. In some cancer processes, efforts to improve patient pathways and patient flows were already in place, even before the CPPs were implemented. This was considered to be a facilitating factor, and one which had a positive impact on the outcome.

*“We already have those things, so we had started with our own little local CCP […] We have been monitoring lead times for a long time. But now with the CPP, it becomes much more concrete and a better flow.” (Brain 1).*

#### Government support

The fact that the policy was developed externally and initiated at the national government level was described as being a positive factor. In some cancer processes, current referral routes and collaboration with other health professional was considered ineffective and poor. The new policy supported and facilitated the work as it created better order and remedy in the process, as illustrated below:

*“No, so the biggest support for me, in my work, it has been that there is a government decision behind this.” (Upper gastro 1).*

### Complexity of joint action between levels in health care

#### Stability of interorganizational collaboration

Collaboration and coordination were important factors for successful implementation. Incorporating an understanding of the multiple actions needed at the different services and levels of the health care system was essential, especially given the non-linear pathways and interdisciplinary character of the policy*.* Each unit’s dependency on linking with other units and care providers became evident. The level of collaboration with other units varied considerably, and this was stated as having a directly negative impact on lead-times for patients in the pathway. Establishing good collaboration with other units was seen as a prerequisite for successful implementation.

*“We are trying to shorten down the lead-times, but some things are outside our control and there is nothing we can do about. There are other organizations involved, radiologists and pathology, and it’s hard even though they know how we work.” (Lung 3).*

#### Differences in capability and sufficiency of resources

The pre-reserved appointments at investigatory units such as radiology, pathology and oncology did not always facilitate the work. This was perceived both by the investigating units themselves and by other units involved in the CPPs. Factors hampering effective collaboration mainly derived from constrains in human, finical and timely resources and paradoxically the desire for shortened waiting times was not always enforceable.

*“In the planning phase of the CPP, when they identified problems [with cancer care], they identified radiology and pathology as bottlenecks, and yet we were not incentivized […] they identified us as bottleneck, and here we are, still the bottlenecks.” (Radiology 6).*

#### Adaptation and changes throughout the process

At the time of the initial implementation, only some aspects of the CPPs were systematically prepared and planned. Rather than routines and activities related to administration and the time-point measurements were continuously changed throughout the implementation, which was described as being both a barrier and a facilitator. Reporting lead-times was one of the core activities in the CPPs, and this was primarily achieved in the existing electronical medical records systems. However, working within disparate systems hampered the possibility of assuring correct coding, monitoring measure points, such as lead-times, and tracing patients. In addition, health care professionals renounced coding, leading to other health care professionals in the chain of care, taking responsibility for registrations retrospectively. For example, an endoscopist has to register the entry point for a pathway, which should have been registered by a GP. Therefore, a new electronic platform, INCA, was launched as a strategy to prevent the recording of misrepresentative data and to enhance uniform coding. The introduction of the platform was perceived as a positive factor, but at the same time, it arrived abruptly and very little information was provided on how to manage the new system.

*“Everyone does not have the same journal system, from the start. I spent a lot of time trying to get in touch with other referral and units [to make sure they report lead-times]. So I’m very happy about this with INCA.” (Gynecology 1).*

#### Unclear demarcation of responsibility

With the launch of the CPPs, a new role of coordinator was established. The purpose of the coordinator’s role was to structure the pathways for the patient and to ensure that lead-times were being upheld. This role had been developed differently at different sites. In some cases, individuals within the organization who had similar roles were assigned to the coordination role. In other settings, individuals outside the organization were recruited specifically for the period of the implementation. There were divergent opinions regarding the impact of the coordinator. Their role was seen as being vital in the CPPs as it contributed to establishing more structure and coherence for the patients with regards to the required investigations and booking them.

*“Now, many have coordinators, which is a great benefit, because then we are striving towards the same goal [...] It becomes more organized and we are able to track the patients.” (Lung 1).*

Nevertheless, difficulties in understanding the responsibilities of the coordinators and what their role encompassed led to confusion for other health care professionals. The coordinator sometimes lacked awareness of what was expected from them and how to relate to their tasks. Some expressed a concern about the future, in how the sustainability of the coordinator’s role could be secured after the implementation was complete and this resource was withdrawn. The coordinator’s tasks would then be delegated to other professionals.

*“I’m thinking about the future, how, I mean I have a job that cannot be liquidated, what will happen later, when there is no funding? Will the clinic outsource these tasks to a medical secretary or other functions?” (Endoscopy 5).*

*“Do not have much insight into what they do. We do not get so much information about what they are doing […] then I do not know how they prioritize their work and what they focus on. Maybe they are working overarching on a higher level, maybe to get those ‘slot-times’.” (Upper gastro 3).*

### Priorities and unintended effects

#### Crowding-out effects

Unintended effects, such as crowding-out effects (situations where lower priority patients are given care before patients who have a higher priority [9]), were highlighted as a risk, especially within endoscopy and radiology. There were divergent views regarding the crowding-out effect of other patient groups, either those without a cancer diagnosis but in need of the same resources as patients with cancer, relapse patients with cancer, or patients with lower priority. It was explained that the CPPs contributed to the subordination of other patients, and, after the implementation, more focus and prioritization was given to those enrolled in the CPPs. Some clinics solely prioritized the patients included in the CPPs.

*“I mean this is the results of lobbyism of course, they have been lobbying to prioritize cancer, patients with suspected cancer, without any consideration whatsoever of the crowding-out effect it might have.” (Endoscopy 1).*

After the CPPs were introduced, shorter waiting times for CPP patients were observed. It was described how the diagnostic process was completed at a faster pace than it had previously, which was considered to be a positive outcome and effect of the CPPs. However, differences in waiting times between CPP patients and other patients was observed.

*“I have noticed a big difference between those who are CPP patients and not CPP patients, CPP patients are treated much faster, usually we have an answer within three days, compared to previously when it took one week.” (Myeloma 2).*

## Discussion

To our knowledge, this paper is the first qualitative study to assess the CPPs and it highlights barriers and facilitators associated with the implementation process of the CPPs, a national policy intended to address challenges related to long waiting times for cancer care in the Swedish setting.

Our results showed that health care professionals involved in, and working within, the CPPs were positive towards the implementation of the new reform. Although most of the health care professionals described observing information gaps during the process, they expressed that the expectations from both professionals outside their own setting as well as the political demands forced them to organize care processes in line with the policy requirements. The fact that they were strongly dependent on each other in order to uphold a care process became clear, which urged for a continuous adaptations of policy activities throughout the implementation.

The urge for change that has been described by respondents can be considered as an effective way to influence the behavior of autonomous caregivers towards the intentions of national policy. For instance, an evaluation of a previous national policy in Sweden found that external pressures in terms of performance bonuses strongly influenced the regional health care providers’ decisions to participate in the policy implementation. Furthermore, peer pressure from other health care organizations, i.e. others implementing the policy, also contributed to the policy implementation in situations where the policy was not perceived to be an effective solution to the local needs [34, 35].

A common perception among respondents was that even though strong efforts were made to achieve the target times in one part of the system, the lack of resources, competence or initiative in another part of the system hindered the processes and created bottlenecks in the system. This strongly influenced the extent to which the actors were motivated or able to act towards the intentions of the CPPs. The effort to introduce the CPPs led to an increased focus in developing integrated care processes and collaboration between different stakeholders within the whole health system [8, 9]. The findings indicate that the collaboration aspect has in many ways been an obstacle, predominantly derived from differences in capability for working according to the structure of the CPPs.

Collaboration was mainly hampered by the differences in availability of resources, access to information, and knowledge about what the new policy encompassed, and how to implement it within the existing structure. As one pathway covers several hospitals, clinics and/or professionals, the awareness and enactment of the new policy was of high relevance for one of the main desired outcomes; shorter waiting times. Policy dissemination and uptake by health professionals about the direct practice and CPPs was perceived by health care professionals as diverse, and, in many sectors of the health care system, especially in primary care, professionals were not aware of the new policy having been put in place. This becomes challenging when the GP often forms the first point of entry into a CPP [8].

Ultimately, in a relatively large setting, such as that of the studied county, health care organizations at one level may be capable of following the time bound-requirements in a pathway, but are paradoxically dependent on other health care providers outside their own setting. The functionality of the CPPs becomes less coherent and uniform when it depends on one or few single units/organizations. The CPP was implemented with an adaptive approach and this made it necessary for the actors in the system to test, learn and adapt during the process which was the case with the introduction of the INCA platform as well as the establishment of a new coordinator role, for whom the responsibilities were unclear. This adaptive and flexible approach in the policy may have contributed to misunderstandings and increased the risk of information and practice gaps.

Nevertheless, flexibility and making room for local adaptations has been proposed to be central for successful implementation. It is about creating fit between the intervention, i.e., the policy, and the local context. In policy development, this implies that not all components should necessarily be pre-developed, and that the implementation is designed to develop in stages, and partly while the policy has first been disseminated to target groups [34]. This might lead stakeholders to perceive that they have been provided with too little information about the changes, however, this tends to change over time [34].

Finally, considering the ethical perspective and the aspect of medical prioritization, the CPPs may, in some respects, imply longer waiting times for other patient and patient groups in need of the same health care resources. As a result, with a focus on cancer, our study found that there is a concern among health care professionals about the potential for horizontal prioritization. Patients affected by serious illness and chronic disease may experience delayed diagnoses and have to wait longer than medically desired in favor of CPP patients. The same applies for those patients whose cancer relapses and patients considered to be of lower priority [4, 8, 9, 22].

### Strengths and limitations

This study has several limitations. The interviewees in our study were predominantly drawn from the public sector, limiting our ability to address divergences between public and private providers. Furthermore, all cancer diagnoses were not included. There is a possibility that health care professionals working in other cancer diagnoses, those not covered in our study, could have provided valuable and different input. The same applies about the choice of county council. In terms of the generalizability, signifying the extent to which findings can be transferred to other settings or patient groups, our study was conducted in one county [32]. The findings may not be applicable in other counties or regions in Sweden, as well as in other countries with different health systems. Nevertheless, one strength is that the informants in our study had a wide range of different roles in the CPP process and represented a variety of cancer diagnoses. Another strength is that this is the first qualitative study on the implementation process of the CPPs. In the presentation of the results we used quotes, which strengthens the validity of our results [29]. Moreover, two researchers conducted the interviews and the analysis was performed with all of the co-authors, allowing for more transparency and diminishing the risk of personal biases [23]. Lastly, the scope of this study is limited to health care professionals; we have not explored the experiences of the patients in the cancer pathways.

## Conclusion

The CPPs were introduced to address challenges with long waiting times and unequal cancer care. Even though the health care professionals had a positive attitude towards the CPPs and saw the value in promoting the standardization of cancer care processes. Our findings show that the implementation of CPPs involve challenges that concern multi-level coordination and collaboration, policy dissemination, and resource constraints. These are challenges that need to be addressed in the implementation of similar pathways in the future. The analysis also showed that the implementation of CPPs risk being accompanied by unintended effects such as longer waiting times for other patients and patient groups in need of the same health care resources. These finding should be further explored in future studies.

## Data Availability

Not applicable. The data will not be shared as ethics approval for the study requires that the transcribed interviews are kept in locked files, accessible only to the researchers.

## Abbreviations

CPP: Cancer patient pathway
GP: General practitioner
RCC: Regional Cancer Center
SCC: Stockholm County council

## References

[1] Siciliani Luigi, Borowitz Michael, Moran Valerie (2013). Waiting Time Policies in the Health Sector.

[2] Viberg N, Forsberg BC, Borowitz M, Molin R (2013). International comparisons of waiting times in health care – Limitations and prospects. Health Policy.

[3] Coleman MP, Forman D, Bryant H, Butler J, Rachet B, Maringe C (2011). Cancer survival in Australia, Canada, Denmark, Norway, Sweden, and the UK, 1995-2007 (the International Cancer Benchmarking Partnership): An analysis of population-based cancer registry data. Lancet.

[4] Robertson S, Adolfsson J, Stattin P, Sjövall A, Winnersjö R, Hanning M (2017). Waiting times for cancer patients in Sweden: A nationwide population-based study. Scand J Public Health.

[5] The National Board of Health and Welfare. Väntetider i cancervården [Waiting times in cancer care]: Socialstyrelsen; 2014.

[6] The National Board of Health and Welfare. Standardiserade vårdförlopp i cancervården [Standardized patient pathways in cancer care]: Socialsyrelsen; 2016.

[7] Government Offices of Sweden. Measures for shorter waiting times in cancer care [Internet]. Stockholm: Regeringskansliet; 2015. Available from: http://www.regeringen.se/regeringens-politik/sjukvard/satsning-pa-kortare-vantetider-inom-cancervarden/ Accessed 14 Nov 2018.

[8] Wilkens J, Thulesius H, Schmidt I, Carlsson C (2016). The 2015 National Cancer Program in Sweden: Introducing standardized care pathways in a decentralized system. Health Policy.

[9] Schmidt I, Thor J, Davidson T, Nilsson F, Carlsson C (2018). The national program on standardized cancer care pathways in Sweden: Observations and findings half way through. Health Policy.

[10] The Norweigan Directorate for Health (2015). Implementering av pakkeforløp for kreft Nasjonal plan for implementering av pakkeforløp for kreft [Implementation of the patient pathways for cancer].

[11] Jensen H, Tørring ML, Olesen F, Overgaard J, Vedsted P (2014). Cancer suspicion in general practice, urgent referral and time to diagnosis: a population-based GP survey and registry study. BMC Cancer.

[12] Ingeman ML, Christensen MB, Bro F, Knudsen ST, Vedsted P (2015). The Danish cancer pathway for patients with serious non-specific symptoms and signs of cancer – A cross-sectional study of patient characteristics and cancer probability. BMC Cancer.

[13] Jensen H, Tørring ML, Vedsted P (2017). Prognostic consequences of implementing cancer patient pathways in Denmark: A comparative cohort study of symptomatic cancer patients in primary care. BMC Cancer.

[14] Probst HB, Hussain ZB, Andersen O (2012). Cancer patient pathways in Denmark as a joint effort between bureaucrats, health professionals and politicians – A national Danish project. Health Policy.

[15] Dodds W, Morgan M, Wolfe C, Raju KS (2004). Implementing the 2-week wait rule for cancer referral in the UK: General practitioners’ views and practices. Eur J Cancer Care.

[16] National Institute for Health and Clinical Excellence (2005). Referral guidelines for suspected cancer.

[17] Jensen H, Vedsted P (2017). Exploration of the possible effect on survival of lead-time associated with implementation of cancer patient pathways among symptomatic first-time cancer patients in Denmark. Cancer Epidemiol.

[18] Sandström B, Borglin G, Nilsson R, Willman A (2011). Promoting the Implementation of Evidence-Based Practice: A Literature Review Focusing on the Role of Nursing Leadership. Worldviews Evid-Based Nurs.

[19] Damschroder LJ, Aron DC, Keith RE, Kirsh SR, Alexander JA, Lowery JC (2009). Fostering implementation of health services research findings into practice: A consolidated framework for advancing implementation science. Implement Sci.

[20] Salamonsen A, Kiil MA, Kristoffersen AE, Stub T, Berntsen GR (2016). “My cancer is not my deepest concern”: Life course disruption influencing patient pathways and health care needs among persons living with colorectal cancer. Patient Prefer Adherence.

[21] The Swedish Association of Local Authorities and Regions. Municipalities, county councils and regions. Sveriges Kommuner och Landsting; 2018 [Internet]. Available from: https://skl.se/tjanster/englishpages/municipalitiescountycouncilsandregions.1088.html Accessed 14 Nov 2018.

[22] The National Board of Health and Welfare. Standardiserade vårdförlopp [Standardized cancer patient pathways] [Internet]. Stockholm: Socialstyrelsen; 2017. Available from: https://www.socialstyrelsen.se/globalassets/sharepoint-dokument/artikelkatalog/ovrigt/2017-11-14.pdf Accessed 14 Nov 2018.

[23] Kvale SBS (2009). Interviews: Learning the craft of qualitative research interviewing.

[24] Polit D, Beck C (2004). Nursing research.

[25] Koontz T, Newig J (2014). From Planning to Implementation: Top-Down and Bottom-Up Approaches for Collaborative Watershed Management. Policy Stud J.

[26] Escoffery C, Lebow-Skelley E, Haardoerfer R, Boing E, Udelson H (2018). A systematic review of adaptations of evidence-based public health interventions globally. Implement Sci.

[27] Council Stockholm County. Healthcare [Internet]. Stockholm: Stockholm läns landsting; 2018. [cited 2018 Nov 15]. Available from: https://www.sll.se/om-landstinget/Information-in-English1/About-Stockholm-County-Council/Healthcare1/ Accessed 14 Nov 2018.

[28] Public Health Agency of Sweden. Folkhälsans utveckling - årsrapport 2017 [Public health development – annual report 2017]. Stockholm: Folkhälsomyndigheten; 2017 Available from: https://www.folkhalsomyndigheten.se/contentassets/9de83d1af6ce4a429e833d3c8d7ccf85/folkhalsans-utveckling-arsrapport-2017-16136-webb2.pdf Accessed 14 Nov 2018.

[29] Patton MQ (2002). Qualitative research and evaluation methods.

[30] Palinkas LA, Horwitz SM, Green CA, Wisdom JP, Duan N, Hoagwood K (2015). Purposeful sampling for qualitative data collection and analysis in mixed method implementation research. Adm Policy Ment Health.

[31] Robinson OC (2014). Sampling in interview-based qualitative research: A theoretical and practical guide. Qual Res Psychol.

[32] Graneheim UH, Lundman B (2004). Qualitative content analysis in nursing research: Concepts, procedures and measures to achieve trustworthiness. Nurse Educ Today.

[33] Braun V, Clarke V (2006). Using thematic analysis in psychology. Qual Res Psychol.

[34] Strehlenert H, Richter-Sundberg L, Nyström ME, Hasson H (2015). Evidence-informed policy formulation and implementation: A comparative case study of two national policies for improving health and social care in Sweden. Implement Sci.

[35] Nyström ME, Strehlenert H, Hansson J, Hasson H (2014). Strategies to facilitate implementation and sustainability of large system transformations: A case study of a national program for improving quality of care for elderly people. BMC Health Serv Res.

